# The TOPCONS web server for consensus prediction of membrane protein topology and signal peptides

**DOI:** 10.1093/nar/gkv485

**Published:** 2015-05-12

**Authors:** Konstantinos D. Tsirigos, Christoph Peters, Nanjiang Shu, Lukas Käll, Arne Elofsson

**Affiliations:** 1 Department of Biochemistry and Biophysics, Stockholm University, 10691 Stockholm, Sweden; 2 Science for Life Laboratory, Stockholm University, Box 1031, 17121 Solna, Sweden; 3 Bioinformatics Infrastructure for Life Sciences (BILS), Stockholm University, Sweden

## Abstract

TOPCONS (http://topcons.net/) is a widely used web server for consensus prediction of membrane protein topology. We hereby present a major update to the server, with some substantial improvements, including the following: (i) TOPCONS can now efficiently separate signal peptides from transmembrane regions. (ii) The server can now differentiate more successfully between globular and membrane proteins. (iii) The server now is even slightly faster, although a much larger database is used to generate the multiple sequence alignments. For most proteins, the final prediction is produced in a matter of seconds. (iv) The user-friendly interface is retained, with the additional feature of submitting batch files and accessing the server programmatically using standard interfaces, making it thus ideal for proteome-wide analyses. Indicatively, the user can now scan the entire human proteome in a few days. (v) For proteins with homology to a known 3D structure, the homology-inferred topology is also displayed. (vi) Finally, the combination of methods currently implemented achieves an overall increase in performance by 4% as compared to the currently available best-scoring methods and TOPCONS is the only method that can identify signal peptides and still maintain a state-of-the-art performance in topology predictions.

## INTRODUCTION

α-helical transmembrane (TM) proteins constitute the most important and well-studied class of membrane proteins. In numbers, they add up to 20–30% of all proteins encoded in a typical genome (1,2). They are involved in cellular recognition, signal transduction and transport of substances through membranes. In addition, membrane proteins facilitate the regulation of the membrane's lipid composition and the formation and maintenance of the shape of membranes and cells (3). They pose a great pharmaceutical interest, since they are targets for a large fraction of all commercial drugs (4–6). The structural and physiochemical properties of these proteins create inherent difficulties in crystallizing and obtaining good quality 3D structures. This leads to their underrepresentation (∼1–2% of all available structures) in the PDB database (7) and dictates the need for developing computational algorithms and tools that will allow for a reliable and fast prediction of their structural and functional features.

A fundamental aspect of the structure of integral proteins is their membrane topology, i.e. the number of TM segments, their position in the protein sequence and their orientation in the membrane. Along these lines, several algorithms for the prediction of α-helical TM protein topology exist, either as single-sequence-based methods (8–16) or with the inclusion of homologous sequences in the prediction process (17–22). In recent years, consensus algorithms that combine the outputs from different predictors have also been developed (23–26). The best methods currently reach an upper limit in their performance of around 70–80% in large data sets (27), but clearly one major problem remains: the separation of signal peptides (SPs) and N-terminal TM regions. This makes proteome-wide predictions of TM proteins less accurate than desirable.

The similarity between signal peptides and N-terminal TM regions is a major challenge for improved topology predictions; because of their similar hydrophobic composition, the cross-prediction of SPs as TM helices and vice versa is quite common (28). Given that, for example, in the human genome, ∼5% of the proteins are predicted to have a signal peptide, it becomes clear that in proteomic analyses it is crucial not to confuse cleaved signal peptides and TM regions (29). Predictors that contain specialized sub-models for signal peptides and TM segments (14,15,18,21,22,30) are thus more useful for proteome-wide analyses. Moreover, the latest version of the most widely used prediction method for detecting signal peptides, SignalP (31), shows an improved performance in discrimination between signal peptides and TM regions; however it cannot predict the topology of TM proteins.

Here, we present an update to the TOPCONS consensus prediction method and its server implementation, including topology prediction methods that can predict signal peptides as well (Philius (15), PolyPhobius (21) and SPOCTOPUS (22), along with OCTOPUS (20) and SCAMPI (9)). This combination of methods results in improved consensus predictions. Moreover, we provide an efficient way for discriminating TM from non-TM proteins, as well as signal peptides from TM regions. Finally, with the increase in the overall speed, it is now possible to scan an entire proteome in few hours/days (depending on its size and the server load), even with the inclusion of homologous sequences in the prediction process.

## MATERIALS AND METHODS

### Data sets used in this study

To benchmark the new version of TOPCONS, we used four different data sets, namely TM-proteins only (‘TM-set’), TM-proteins that also have a cleavable signal peptide in their N-terminal (‘SP+TM-set’), globular proteins (‘Globular-set’) and secreted proteins that only have a signal peptide and no membrane regions (‘Globular+SP-set’). The TM proteins were initially retrieved from the PDBTM database (32) and mapped to their respective UniProt (33) sequences using the SIFTS (34) database. For topology assignment, we combined different sources (PDBTM, OPM (35), TOPDB (36) and UniProt), along with manual inspection in some spurious cases. The other three data sets originated from the TOPDB database and the SignalP4 method. In order to have a fair evaluation, we performed a 30% homology reduction using BLASTclust (37) on all proteins together and were left with 313 proteins in the ‘TM-set’, 752 in the ‘SP+TM’, 3597 in the ‘Globular’ and 2194 in the ‘Globular+SP’ set. In this way, a more representative view of a proteome can be studied. All annotated data sets are available for download from the website.

### The TOPCONS algorithm

**Figure 1.**
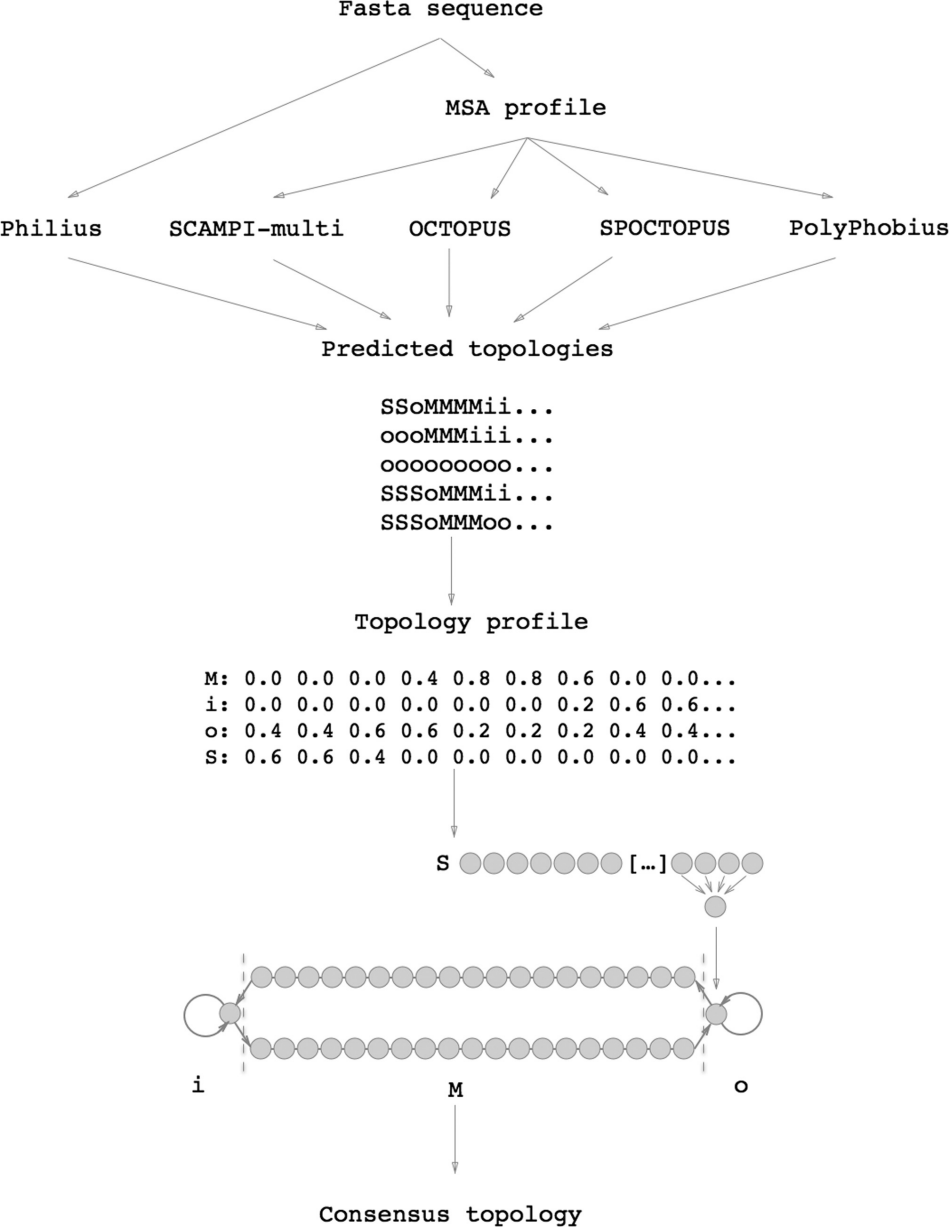
The TOPCONS workflow: four of the topology predictors (OCTOPUS, PolyPhobius, SPOCTOPUS and SCAMPI) use an MSA-derived sequence profile as input, whereas the fifth method (Philius) only requires the protein sequence. The topology predictions are used to construct a topology profile, which is fed into the TOPCONS Hidden Markov Model and the final consensus topology is created.

The core algorithm of TOPCONS remains the same as the earlier implementation, with the addition of a signal peptide module (see Figure 1). The topology predictions from the five sub-methods used (OCTOPUS, Philius, PolyPhobius, SCAMPI and SPOCTOPUS) are combined into a topology profile, where each residue is represented by four values, corresponding to the fraction of methods that predict that particular residue to belong to a signal peptide (S), a membrane region (M) or the membrane-inside and outside (i and o, respectively). A dynamic programming algorithm, represented as a Hidden Markov Model, that has an alphabet consisting of the characters ‘S’, ‘M’, ‘i’ and ‘o’ processes the resulting profile. The final topology corresponds to the highest scoring state path through this model using a Viterbi-like algorithm. In each state, the emission score for the structural category modeled by that state (S, i, o or M) is equal to 1.0 and for all other structural categories it equals to 0.0. All transition probabilities are equal to 1.0. Thus, the final prediction equals to the state path with the highest geometric mean score with respect to the topology profile and the grammar of the model, and no training of the model is necessary. In addition, the biological hydrophobicity scale (38) is used to predict the free energy of membrane insertion for a window of 21 amino acids centered on each position in the sequence.

### Shortening the process time

**Figure 2.**
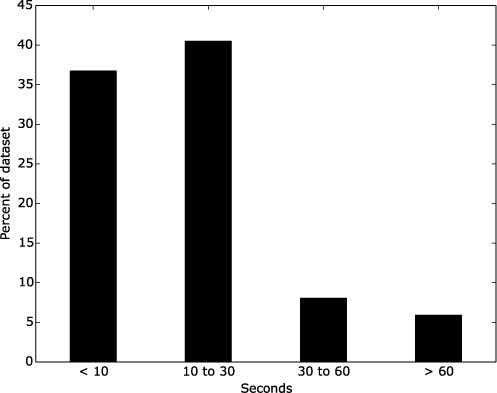
Distribution of time (in seconds) required for processing the proteins in all data sets we used in the benchmark. The increase in speed is substantial, since almost 80% of all proteins in total took less than 30 seconds.

Traditionally, the best performance in membrane protein topology predictions is achieved by using a profile. The best profiles are obtained by searching a large database, such as UniRef (39). However, given the rapid increase in database sizes, such a search often takes several minutes using a single computer. This is not optimal for the experience of a web server, where the user aims for a prompt response. In the previous TOPCONS configuration, using a smaller database consisting only of membrane proteins circumvented this problem. This did not significantly affect the predictions of topologies in membrane proteins. However, many non-membrane proteins had very few related protein hits in a PSI-BLAST (40) query, and thus some membrane regions were erroneously predicted in them. In the new version of TOPCONS, we have switched to a two-step pipeline: first we scan the query sequence(s) against Pfam (41) and then all full-length sequences are used to create a query-specific database which is further scanned for homologous proteins. Because the domain database and the number of hits found are both much less than all proteins in UniProt or even UniRef, this search is much faster. Moreover, since almost all proteins have domain hits, the resulting profiles are virtually identical to the ones found when searching the entire database. In this way, we combine both the speed in the earlier version of TOPCONS using a small membrane protein-containing database with the ability to separate membrane and non-membrane proteins obtained when using a much larger database. If no hits can be retrieved with the afore-mentioned procedure, we scan the CDD database (42) using the ‘hmmscan’ program from the HMMER3 suite (43). This step is more time consuming, but we anticipate it will not occur very frequently. In the benchmark data sets we used, there were only 350 proteins (∼5% of the total proteins) that had no hits and we had to use the fallback to the CDD alternative. For an overview of the speed of processing queries see Figure 2. The vast amount of proteins that we tested were processed in less than half a minute, whereas only around 6% of them required more than a minute to output the final prediction. The median time to process the queries in our data sets on a 4-core machine was ∼11 seconds. However, the computational times might occasionally be longer due to heavy demand on the server.

## RESULTS

### Benchmark results

#### Membrane protein topology predictions

**Figure 3.**
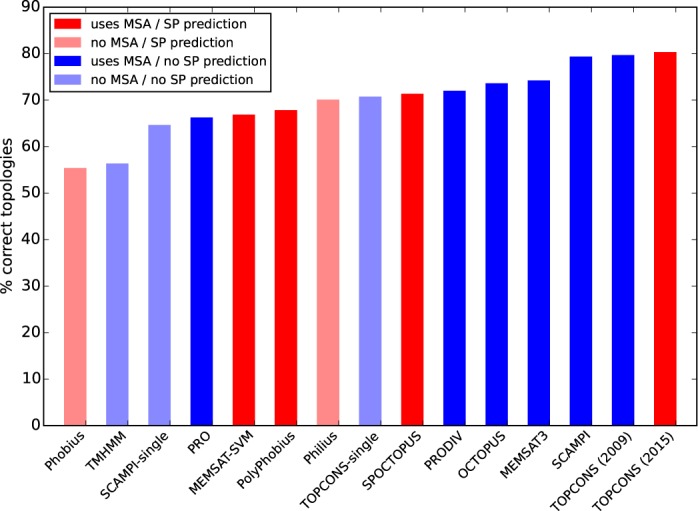
Comparison of the topology prediction accuracy of the current TOPCONS implementation versus other topology prediction methods. Note that the performance drops for all predictors that predict both signal peptides and TM regions as opposed to methods specifically designed to predict the topology of membrane proteins.

In Figure 3, the fractions of correctly predicted membrane protein topologies for several methods are shown. In agreement with earlier studies, the best methods predict about 80% of the topologies correctly. Further, it is clear that modern methods which use multiple sequence alignments are superior to older methods and methods that do not use multiple sequence alignments. However, such methods (colored in dark blue in Figure 3), including the older version of TOPCONS, are not designed for proteome-wide analyses because they are mainly focused on correctly predicting the topology of TM proteins. Further, they cannot differentiate between a signal peptide and an N-terminal TM region, thus the number of observed cross-predictions is extremely high. We can conclude that the current implementation of TOPCONS shows the best performance for topology predictions and is the only among the best performing methods that also predicts signal peptides. For other predictors, when we look at their performance, we note that methods that are designed to predict both the presence of a signal peptide and the topology of membrane proteins do not perform as well as methods that do not. For instance, MEMSAT-SVM only predicts 67% of the topologies correctly (TM-set), whereas the related method MEMSAT3 achieves a performance of 74%.

In Table 1, we show the performance on the four different sets, including only methods that can be actually evaluated on all of them (i.e. the methods that can predict signal peptides). From the table it is clear that the new implementation of TOPCONS shows an important improvement in topology predictions for membrane proteins: if we only focus on membrane proteins with no signal peptide, TOPCONS’ performance is 9% greater than SPOCTOPUS which ranks second. If we further combine the results for both the ‘TM’ and the ‘SP+TM’ sets, we see that the average performance for TOPCONS is 80%, while, the second best, SPOCTOPUS, reaches 75% (5% increase in correct topology prediction).

**Table 1.** Performance of several topology prediction methods, appropriate for whole-proteome scanning, along with the current TOPCONS implementation

Method	MSA	TM	SP+TM	Globular	Globular+SP	Overall
TOPCONS	+	80%	80%	97%	91%	87%
MEMSAT-SVM	+	67%	52%	88%	0.0%	52%
Philius	−	70%	75%	94%	94%	83%
Phobius	−	55%	83%	95%	94%	82%
PolyPhobius	+	68%	64%	95%	85%	78%
SPOCTOPUS	+	71%	78%	78%	79%	76%

For the TM-set, the correct topology should have the correct number of TM regions at approximately correct locations (overlap of at least five residues) and the correct location of the N and C-termini; for the SP+TM-set we also require the prediction of a signal peptide in the N-terminal of the protein sequence; for the Globular-set we require that no membrane regions and no signal peptides are predicted in order for a prediction to be considered as correct; finally, for the Globular+SP set, the predictor should only predict the presence of a signal peptide in the sequence.

#### Whole-proteome scanning

In Table 1, the performance of topology prediction methods that also predict the presence of signal peptides and therefore are more useful for scanning an entire proteome is listed. Using all four sets, we can estimate the performance of a proteome-wide scan, since all types of proteins are covered. It should be noted that, in a genome, most encoded proteins are globular and therefore it might be more important to have a high specificity for these proteins. All in all, TOPCONS reaches an accuracy of 87%, followed by Philius and Phobius with 83 and 82%, respectively. TOPCONS is superior to all other methods in the ‘TM’ and ‘Globular’ sets and has close to the best performance in the ‘SP+TM’ set. Philius and Phobius perform better in the data set that only contains signal peptides (94% on the ‘Globular+SP-set’), while TOPCONS correctly identifies 91% of the proteins in this case. In comparison to the other methods, more signal peptides are missed and more proteins contain erroneously predicted TM regions (see Table 2 and Supplementary Table S1 for the other methods used in the benchmark). Interestingly, a similar lower performance on the ‘Globular+SP-set’ can be seen for SPOCTOPUS, MEMSAT-SVM and PolyPhobius, indicating that possibly the identification of signal peptide cleavage sites is superior in methods not using multiple sequence alignments. Surprisingly enough, MEMSAT-SVM in this set cannot predict the presence of a signal peptide without, at the same time, predicting one or more TM helices in the protein sequence. In Table 3 (and Supplementary Table S2), the performance of TOPCONS (and the other methods) regarding protein classification in the four different categories is shown. We observe that TOPCONS is very accurate in correctly identifying a non-membrane protein (97%), however, it is clear that the difficulty in differentiating a signal peptide from an N-terminal TM region still holds; in 12% of the proteins in the ‘SP+TM’ set, the signal peptide is misclassified as TM helix. Further, in 4% of the proteins in the ‘TM*-*set’, we obtain a falsely predicted signal peptide, whereas in 7% of the proteins that have only a signal peptide (‘Globular+SP-set’), extra TM regions are predicted in the non-membrane regions. Philius, which is the only of the other methods that is relatively better in classification than TOPCONS (with the exception of ‘Globular­-set’), has the drawback that it makes a lot of wrong topology predictions (it mostly predicts inverted topologies), which is a crucial fact in membrane protein topology prediction.

**Table 2.** Confusion matrix for all type of errors that TOPCONS makes

Data set	Correct prediction	Wrong topology	TM → SP or SP → TM	TM → non-TM or non-TM → TM	non-TM → SP or SP → non-TM
TM	80%	16%	2.6%	0.9%	--
SP+TM	80%	7.0%	13%	--	0.0%
Globular+SP	91%	--	7.2%	--	1.8%
Globular	97%	--	--	1.5%	1.5%

Correct prediction: requires that both the classification and the topology of the given protein are correct; Wrong topology: the classification is correct but the overall topology is not (e.g. extra predicted TM helices in non-membrane regions); TM → SP or SP → TM: the N-terminal TM helix is wrongly assigned as a signal peptide or vice versa; TM → non-TM: a TM protein is classified as non-TM protein or vice versa; SP → non-TM: a protein with a signal peptide or a protein with a signal peptide and transmembrane region(s) is classified as non-TM protein or vice versa.

**Table 3.** Confusion matrix for classification of proteins in each of the data sets using the TOPCONS algorithm

Data set	TM	SP+TM	Globular+SP	Globular
TM	95%	3.0%	1.0%	1.0%
SP+TM	12%	86%	2.0%	0.0%
Globular+SP	1.0%	6.0%	91%	2.0%
Globular	1.0%	0.0%	2.0%	97%

Each row shows the number of proteins in one class that is categorized to each of the four classes (transmembrane, signal peptide and transmembrane, only signal peptide and globular). It can be seen that the vast majority of wrong classifications are between transmembrane regions and signal peptides.

### The TOPCONS web server

In the updated version of the TOPCONS web server (http://topcons.net/), we have maintained the already existing user-friendly environment. Now, the input to the server can either be one FASTA-formatted amino acid sequence or a file with multiple sequences that will be processed in due time. For cases of proteins with a determined 3D-structure, we also provide the topology based on the respective PDB entry (topology information as included in PDBTM (32) database). Further, if the query protein is found to bear a significant similarity to a protein with a 3D-structure, then, based on their pairwise alignment, we assign the N-terminal and TM segment boundaries on it. This should be useful for analysis of potential variation of topologies within a protein family.

**Figure 4.**
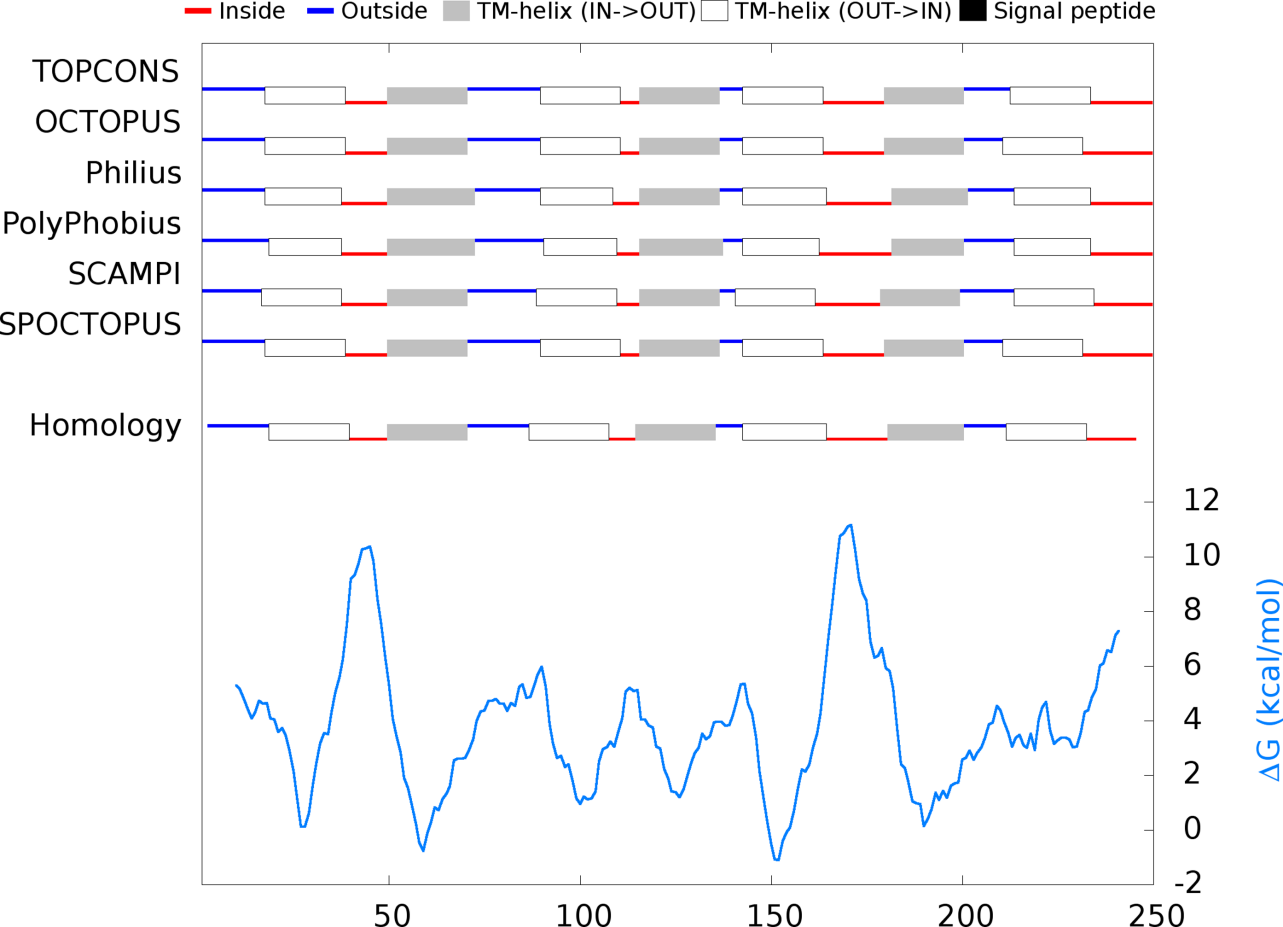
Example output from the TOPCONS web server, based on the Bacteriorhodopsin sequence from *Halobacterium* sp. (UniProt-ID: BACR_HALS4). Topology predicted by TOPCONS, the individual methods and predicted ΔG values across the sequence.

An example output of the web server is shown in Figure 4. The results are presented to the user graphically on the screen but can also be downloaded in plain-text format and/or sent by email if provided. Given the increase in speed process of the new TOPCONS version and the addition of an efficient queuing system, it is now possible to submit even entire proteomes to the server. For instance, scanning the entire human proteome now takes a few days on a dedicated single 4-core machine. Should demands rise in the future, we hope to be able to attract funding to increase the hardware capacity. To facilitate for proteome-wide assignments, we have also developed a standard WSDL interface for programmatic use of the web-server.

## AVAILABILITY

http://topcons.net/

## SUPPLEMENTARY DATA

Supplementary Data are available at NAR Online.

SUPPLEMENTARY DATA

## References

[1] Krogh A., Larsson B., von Heijne G., Sonnhammer E.L. (2001). Predicting transmembrane protein topology with a hidden Markov model: application to complete genomes. J. Mol. Biol..

[2] Wallin E., von Heijne G. (1998). Genome-wide analysis of integral membrane proteins from eubacterial, archaean, and eukaryotic organisms. Protein Sci..

[3] von Heijne G. (2007). The membrane protein universe: what's out there and why bother. J. Inter. Med..

[4] Bakheet T.M., Doig A.J. (2009). Properties and identification of human protein drug targets. Bioinformatics.

[5] Davey J. (2004). G-protein-coupled receptors: new approaches to maximise the impact of GPCRS in drug discovery. Expert Opin. Ther. Targets.

[6] Yildirim M.A., Goh K.I., Cusick M.E., Barabasi A.L., Vidal M. (2007). Drug-target network. Nat. Biotechnol..

[7] Berman H.M., Battistuz T., Bhat T.N., Bluhm W.F., Bourne P.E., Burkhardt K., Feng Z., Gilliland G.L., Iype L., Jain S. (2002). The Protein Data Bank. Acta Crystallogr. D Biol. Crystallogr..

[8] Claros M.G., von Heijne G. (1994). TopPred II: an improved software for membrane protein structure predictions. Comput. Appl. Biosci..

[9] Bernsel A., Viklund H., Falk J., Lindahl E., von Heijne G., Elofsson A. (2008). Prediction of membrane-protein topology from first principles. Proc. Natl. Acad. Sci. U.S.A..

[10] Jones D.T., Taylor W.R., Thornton J.M. (1994). A model recognition approach to the prediction of all-helical membrane protein structure and topology. Biochemistry.

[11] Rost B., Fariselli P., Casadio R. (1996). Topology prediction for helical transmembrane proteins at 86% accuracy. Protein Sci..

[12] Tusnady G.E., Simon I. (2001). The HMMTOP transmembrane topology prediction server. Bioinformatics.

[13] Bagos P.G., Liakopoulos T.D., Hamodrakas S.J. (2006). Algorithms for incorporating prior topological information in HMMs: application to transmembrane proteins. BMC Bioinformatics.

[14] Kall L., Krogh A., Sonnhammer E.L. (2004). A combined transmembrane topology and signal peptide prediction method. J. Mol. Biol..

[15] Reynolds S.M., Kall L., Riffle M.E., Bilmes J.A., Noble W.S. (2008). Transmembrane topology and signal peptide prediction using dynamic bayesian networks. PLoS. Comput. Biol..

[16] Tsaousis G.N., Bagos P.G., Hamodrakas S.J. (2014). HMMpTM: improving transmembrane protein topology prediction using phosphorylation and glycosylation site prediction. Biochim. Biophys. Acta.

[17] Jones D.T. (2007). Improving the accuracy of transmembrane protein topology prediction using evolutionary information. Bioinformatics.

[18] Nugent T., Jones D.T. (2009). Transmembrane protein topology prediction using support vector machines. BMC Bioinformatics.

[19] Viklund H., Elofsson A. (2004). Best alpha-helical transmembrane protein topology predictions are achieved using hidden Markov models and evolutionary information. Protein Sci..

[20] Viklund H., Elofsson A. (2008). OCTOPUS: improving topology prediction by two-track ANN-based preference scores and an extended topological grammar. Bioinformatics.

[21] Kall L., Krogh A., Sonnhammer E.L. (2005). An HMM posterior decoder for sequence feature prediction that includes homology information. Bioinformatics.

[22] Viklund H., Bernsel A., Skwark M., Elofsson A. (2008). SPOCTOPUS: a combined predictor of signal peptides and membrane protein topology. Bioinformatics.

[23] Bernsel A., Viklund H., Hennerdal A., Elofsson A. (2009). TOPCONS: consensus prediction of membrane protein topology. Nucleic Acids Res..

[24] Arai M., Mitsuke H., Ikeda M., Xia J.X., Kikuchi T., Satake M., Shimizu T. (2004). ConPred II: a consensus prediction method for obtaining transmembrane topology models with high reliability. Nucleic Acids Res..

[25] Klammer M., Messina D.N., Schmitt T., Sonnhammer E.L. (2009). MetaTM - a consensus method for transmembrane protein topology prediction. BMC Bioinformatics.

[26] Hennerdal A., Elofsson A. (2011). Rapid membrane protein topology prediction. Bioinformatics.

[27] Tsirigos K.D., Hennerdal A., Kall L., Elofsson A. (2012). A guideline to proteome-wide alpha-helical membrane protein topology predictions. Proteomics.

[28] Lao D.M., Arai M., Ikeda M., Shimizu T. (2002). The presence of signal peptide significantly affects transmembrane topology prediction. Bioinformatics.

[29] Kall L. (2010). Prediction of transmembrane topology and signal peptide given a protein's amino acid sequence. Methods Mol. Biol..

[30] Kall L., Krogh A., Sonnhammer E.L. (2007). Advantages of combined transmembrane topology and signal peptide prediction–the Phobius web server. Nucleic Acids Res..

[31] Petersen T.N., Brunak S., von Heijne G., Nielsen H. (2011). SignalP 4.0: discriminating signal peptides from transmembrane regions. Nat. Methods.

[32] Kozma D., Simon I., Tusnady G.E. (2013). PDBTM: Protein Data Bank of transmembrane proteins after 8 years. Nucleic Acids Res..

[33] UniProt Consortium (2014). Activities at the Universal Protein Resource (UniProt). Nucleic Acids Res..

[34] Velankar S., Dana J.M., Jacobsen J., van Ginkel G., Gane P.J., Luo J., Oldfield T.J., O'Donovan C., Martin M.J., Kleywegt G.J. (2013). SIFTS: Structure Integration with Function, Taxonomy and Sequences resource. Nucleic Acids Res..

[35] Lomize M.A., Lomize A.L., Pogozheva I.D., Mosberg H.I. (2006). OPM: orientations of proteins in membranes database. Bioinformatics.

[36] Dobson L., Lango T., Remenyi I., Tusnady G.E. (2015). Expediting topology data gathering for the TOPDB database. Nucleic Acids Res..

[37] Altschul S.F., Gish W., Miller W., Myers E.W., Lipman D.J. (1990). Basic local alignment search tool. J. Mol. Biol..

[38] Hessa T., Meindl-Beinker N.M., Bernsel A., Kim H., Sato Y., Lerch-Bader M., Nilsson I., White S.H., von Heijne G. (2007). Molecular code for transmembrane-helix recognition by the Sec61 translocon. Nature.

[39] Suzek B.E., Huang H., McGarvey P., Mazumder R., Wu C.H. (2007). UniRef: comprehensive and non-redundant UniProt reference clusters. Bioinformatics.

[40] Altschul S.F., Madden T.L., Schaffer A.A., Zhang J., Zhang Z., Miller W., Lipman D.J. (1997). Gapped BLAST and PSI-BLAST: a new generation of protein database search programs. Nucleic Acids Res..

[41] Finn R.D., Bateman A., Clements J., Coggill P., Eberhardt R.Y., Eddy S.R., Heger A., Hetherington K., Holm L., Mistry J. (2014). Pfam: the protein families database. Nucleic Acids Res..

[42] Marchler-Bauer A., Zheng C., Chitsaz F., Derbyshire M.K., Geer L.Y., Geer R.C., Gonzales N.R., Gwadz M., Hurwitz D.I., Lanczycki C.J. (2013). CDD: conserved domains and protein three-dimensional structure. Nucleic Acids Res..

[43] Eddy S.R. (2011). Accelerated Profile HMM Searches. PLoS. Comput. Biol..

