# Supplementary material for: The TOPCONS web server for consensus prediction of membrane protein topology and signal peptides
Source: Nucleic Acids Res. 2015 May 12;43(Web Server issue):W401–7. doi: 10.1093/nar/gkv485 (PMC4489233; doi:10.1093/nar/gkv485)
Supplement: SUPPLEMENTARY DATA [file supp_43_W1_W401__index.html]

The TOPCONS web server for consensus prediction of membrane protein topology and signal peptides — The TOPCONS web server for consensus prediction of membrane protein topology and signal peptides — SUPPLEMENTARY DATA 

# The TOPCONS web server for consensus prediction of membrane protein topology and signal peptides

## SUPPLEMENTARY DATA

- SUPPLEMENTARY DATA
