# Supplementary material for: The TOPCONS web server for consensus prediction of membrane protein topology and signal peptides
Source: Nucleic Acids Res. 2015 May 12;43(Web Server issue):W401–7. doi: 10.1093/nar/gkv485 (PMC4489233; doi:10.1093/nar/gkv485)
Supplement: SUPPLEMENTARY DATA [file supp_gkv485_nar-00363-web-b-2015-File006.pdf]

Supplementary tables

(a) MEMSAT-SVM

Dataset	Correct prediction	Wrong topology	TM → SP or SP → TM	TM → non-TM Or Non-TM → TM	non-TM → SP or SP → non-TM
TM	67%	22%	11%	0.0%	-
SP+TM	52%	11%	37%	-	0.0%
Globular+SP	0.0%	-	45%	-	55%
Globular	88%	-	-	12%	0.0%

(b) Philius

Dataset	Correct prediction	Wrong topology	TM → SP or SP → TM	TM → non-TM Or Non-TM → TM	non-TM → SP or SP → non-TM
TM	70%	27%	1.5%	1.5%	-
SP+TM	75%	16%	9.0%	-	0.0%
Globular+SP	94%	-	3.5%	-	2.5%
Globular	94%	-	-	2.4%	3.6%

(c) Phobius

Dataset	Correct prediction	Wrong topology	TM → SP or SP → TM	TM → non-TM Or Non-TM → TM	non-TM → SP or SP → non-TM
TM	55%	35%	8.7%	1.3%	-

SP+TM	83%	8.4%	8.6%	-	0.0%
Globular+SP	94%	-	5.0%	-	1.0%
Globular	95%	-	-	2.7%	2.3%

(d) PolyPhobius

Dataset	Correct prediction	Wrong topology	TM → SP or SP → TM	TM → non-TM Or Non-TM → TM	non-TM → SP or SP → non-TM
TM	68%	26%	5.1%	0.9%	-
SP+TM	64%	6.0%	30%	-	0
Globular+SP	85%	-	4.0%	-	11%
Globular	95%	-	-	3.8%	1.2%

(e) SPOCTOPUS

Dataset	Correct prediction	Wrong topology	TM → SP or SP → TM	TM → non-TM Or Non-TM → TM	non-TM → SP or SP → non-TM
TM	71%	20%	7.9%	1.1%	-
SP+TM	78%	11%	11%	-	0.0%
Globular+SP	79%	-	19%	-	2.0%
Globular	78%	-	-	21%	1.0%

Supplementary table 1. Confusion matrix for types of errors made by each of the evaluated prediction algorithms in all datasets.

(a) MEMSAT-SVM

Dataset	TM	SP+TM	Globular+SP	Globular
TM	89%	10%	0.0%	1.0%
SP+TM	36%	61%	0.0%	3.0%
Globular+SP	18%	26%	0.0%	56%
Globular	11%	0.9%	0.0%	88%

(b) Philius

Dataset	TM	SP+TM	Globular+SP	Globular
TM	96%	2.0%	2.0%	0.0%
SP+TM	8.0%	84%	8.0%	0.0%
Globular+SP	1.0%	4.0%	94%	1.0%
Globular	2.0%	0.0%	4.0%	94%

(c) Phobius

Dataset	TM	SP+TM	Globular+SP	Globular
TM	90%	8.0%	1.0%	1.0%
SP+TM	8.0%	90%	2.0%	0.0%
Globular+SP	1.0%	4.0%	94%	1.0%
Globular	3.0%	0.0%	2.0%	95%

(d) PolyPhobius

Dataset	TM	SP+TM	Globular+SP	Globular
TM	94%	5.0%	1.0%	0.0%
SP+TM	30%	68%	1.0%	1.0%
Globular+SP	2.0%	3.0%	85%	10%
Globular	4.0%	0.0%	1.0%	95%

(e) SPOCTOPUS

Dataset	TM	SP+TM	Globular+SP	Globular
TM	91%	8.0%	1.0%	0.0%
SP+TM	10%	89%	1.0%	0.0%
Globular+SP	2.0%	17%	79%	2.0%
Globular	20%	0.0%	2.0%	78%

Supplementary table 2. Confusion matrix for classification of proteins in each of the datasets using the algorithms in the benchmark.
